# Evaluating the Efficacy and Safety of Deoxycholic Acid Injection in Reduction of Flank Fat

**DOI:** 10.1111/jocd.70436

**Published:** 2025-09-01

**Authors:** Navid Namakizadeh Esfahani, Faezeh Khorasanizadeh, Amirhoushang Ehsani, Maryam Nasimi, Hamidreza Mahmoudi, Hamidreza Kafi, Araz Sabzvari, Hoshyar Gholami, Keysan Saeedi, Kamran Balighi

**Affiliations:** ^1^ Department of Dermatology, Razi Hospital Tehran University of Medical Sciences Tehran Iran; ^2^ Autoimmune Bullous Diseases Research Center Tehran University of Medical Sciences Tehran Iran; ^3^ Advanced Diagnostic and Interventional Radiology Research Center (ADIR) Tehran University of Medical Sciences Tehran Iran; ^4^ Department of Radiology, Razi Hospital Tehran University of Medical Sciences Tehran Iran; ^5^ Medical Department Orchid Pharmed Company Tehran Iran; ^6^ CinnaGen Medical Biotechnology Research Center Alborz University of Medical Sciences Karaj Iran; ^7^ Espad Pharmed Darou Company Tehran Iran; ^8^ Department of Industrial Engineering Amirkabir University of Technology (Tehran Polytechnic) Tehran Iran

**Keywords:** fat injections, lipolysis, noninvasive

## Abstract

**Background:**

Deoxycholic acid (DCA) injection is a noninvasive method for localized fat reduction, offering lower risks compared to invasive techniques. This study evaluates the safety and efficacy of DCA injections for reducing flank fat.

**Aims:**

We assessed the efficacy and safety of DCA injections for flank fat reduction over a 12‐week period using imaging, anthropometric data, Global Aesthetic Improvement Scale (GAIS) scoring, participant satisfaction, and standardized adverse event reporting.

**Patients/Methods:**

Adults aged 21–65 years with mild to moderate flank fat (≥ 2 cm) received two sessions of 10 mg/mL DCA injections, 6 weeks apart. Standardized ultrasound and caliper measurements were performed prior to each session. Injections were administered at 0.1–0.15 mL per 1 × 1 cm^2^ area, with a maximum of 2 mL per flank per session, following standardized marking and injection protocols.

**Results:**

Thirty participants (mean age 34.9 years; 86.7% female) completed the study. By Week 12, 86.7% showed aesthetic improvement based on GAIS score, with significant reductions in waist circumference and fat thickness (measured using caliper and ultrasound; *p* < 0.001). The procedure was generally safe, with most adverse events being mild to moderate and limited to the injection site.

**Conclusion:**

DCA injections effectively reduced localized flank fat after two treatment sessions, resulting in significant aesthetic improvements. Adverse events were mostly mild and manageable, with no serious complications.

## 1 Introduction

With the increasing demand for body contouring, noninvasive and minimally invasive fat reduction techniques have gained significant popularity over the past decade [1]. Localized fat deposits commonly accumulate in the abdomen, flanks, thighs, inner knees, and arms. While dietary modifications, physical activity, and bariatric surgery may be effective for overall obesity management, aesthetic procedures remain necessary for eliminating localized fat deposits resistant to conventional interventions, particularly in the flanks and abdomen [2].

Liposuction is the most widely used procedure for removing excess localized fat; however, as a surgical and invasive technique, it carries risks such as pain, infection, prolonged recovery, scarring, hematoma, deep vein thrombosis, pulmonary embolism, and anesthesia‐related complications. These adverse effects and extended recovery periods have led participants to seek less invasive alternatives for body contouring. Noninvasive fat reduction methods currently include low‐level laser therapy, radiofrequency, ultrasound, infrared light, cryolipolysis, and injectable fat‐reducing solutions [2].

Among available treatment options, minimally invasive procedures with fewer complications are particularly appealing for participants desiring localized fat reduction for body contouring. Several clinical trials have investigated subcutaneous injections of phosphatidylcholine and bile salts, such as sodium deoxycholate, for fat reduction, contributing to the growing popularity of injectable lipolysis. Studies indicate that deoxycholic acid (DCA) is the component responsible for clinically significant fat reduction [3].

DCA is a bile acid that emulsifies dietary fat in the intestines and induces nonspecific adipocytolysis by disrupting adipocyte membranes. Purified synthetic DCA was the first pharmacological intervention approved by the FDA for submental fat reduction [4, 5].

Given the promising effects of injectable DCA for localized reduction, along with the limitations of surgical and invasive techniques due to associated complications, participant reluctance, and the need for further safety and efficacy studies, this study aims to evaluate the effectiveness and safety of DCA injections, Embella (manufactured by Espad Pharmed Darou Company), for flank fat reduction in a well‐controlled clinical setting.

## 2 Methods

### 2.1 Study Design and Participants

This single‐arm, open‐label clinical trial was conducted at Razi Hospital, Tehran University of Medical Sciences, between August and December 2024, with ethical approval granted under code IR.TUMS.MEDICINE.REC.1402.700 and registered under clinical trial code IRCT20240416061507N1. The study was conducted according to Good Clinical Practice (GCP) guidelines and the Declaration of Helsinki. Also, written informed consent was obtained from all participants before enrollment.

Eligible participants were adults aged 21–65 with clinically confirmed mild to moderate flank fat, defined as a minimum of 2 cm in thickness as measured by calipers and verified by ultrasound. Participants were required to seek aesthetic improvement and were willing to complete the entire treatment protocol. All participants provided informed consent and agreed not to undergo any additional flank‐related procedures during the study.

Exclusion criteria included any history of flank fat reduction treatments within the past 12 months, plans for future weight control interventions during the study period, or medical conditions that could affect study outcomes. These conditions included coagulation disorders, active infections at the treatment site, known hypersensitivity to any components used in the study, disorders associated with abnormal fat metabolism, dermatological diseases impairing wound healing, obesity (Body mass index > 30 or waist circumference > 105 cm), or other significant comorbidities.

### 2.2 Intervention Protocol

Before treatment, a radiologist performed an ultrasound examination to assess flank fat thickness, and measurements were also taken using a caliper. Eligible participants received 0.1–0.15 mL injections of Embella (10 mg/mL DCA solution, manufactured by Espad Pharmed Darou Company) per 1 × 1 cm^2^ treatment area, with a maximum injection volume of 2 mL per side per session using standard 1 mL insulin syringes equipped with 27‐gauge, 1.3 cm needles. Treatments were administered every 6 weeks for a total of two sessions (Baseline and Week 6). Ultrasound and caliper measurements were repeated at each visit before injection.

To ensure consistent and reproducible injection sites, a standardized marking technique was employed:

1. The posterior axillary line was marked bilaterally.
2. The maximum fat bulge, identified by the participant and physician, was selected along this line.
3. This point served as the center of a 1 × 1 cm injection grid.
4. To standardize injections in subsequent sessions, a perpendicular reference line was drawn from the Anterior Superior Iliac Spine (ASIS) to the posterior axillary line, and the distance from their intersection to the injection site was recorded for precise localization.

**FIGURE 1:**
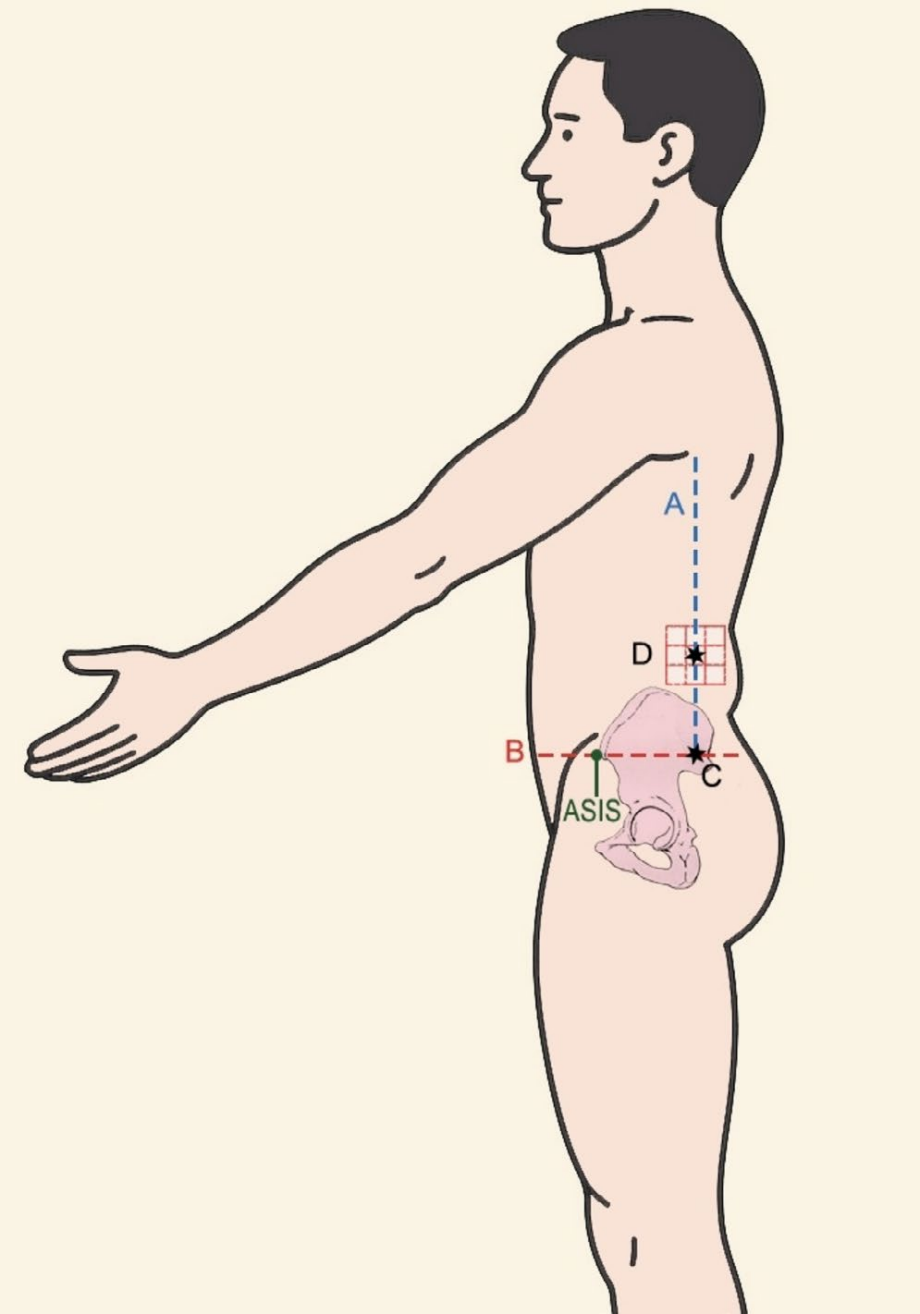
Standardized marking technique to ensure consistent injection sites is illustrated in this schematic image. A vertical line (Line A) is drawn along the posterior axillary line, followed by a perpendicular horizontal line (Line B) intersecting Line A and passing through the anterior superior iliac spine (ASIS). The point of maximal bulging of the flank fat along the posterior axillary line is identified and marked as Point D, representing the center of the injection zone. The distance between the intersection of Lines A and B (Point C) and the injection point (Point D) is recorded to facilitate accurate sonographic assessments and reproducible injections in subsequent treatment sessions.

The standardized marking technique used to ensure consistent injection site localization is illustrated in Figure 1, as depicted in the schematic image.

To minimize discomfort, topical lidocaine was applied to the injection site for 20 min before treatment, and an ice pack was intermittently applied for 30 min postinjection to reduce swelling. Participants were monitored for at least 30 min posttreatment for potential adverse reactions.

### 2.3 Outcome Assessment

At the baseline visit, all participants underwent a comprehensive evaluation, including ultrasonographic measurement of dermal and hypodermal thickness on both flanks, caliper‐based assessment of flank fat on each side, body weight, and measurements of waist and bilateral thigh circumferences using a flexible tape measure. Standardized photographs were also taken at this stage. These assessments were repeated at the follow‐up visits conducted at weeks 6 and 12.

From the second session, participant satisfaction was assessed using a 10‐point Likert scale questionnaire. In order to assess the safety outcome, any adverse event (AE) was recorded using a standardized reporting form that documented the severity, seriousness, and potential relationship to the intervention. AEs were assessed at all visits, and all reported events were classified as mild, moderate, or severe. Furthermore, the seriousness of AEs was determined in accordance with the International Council for Harmonization (ICH‐E2B) guidelines, while the causality was assessed based on the World Health Organization (WHO) criteria.

**FIGURE 2:**
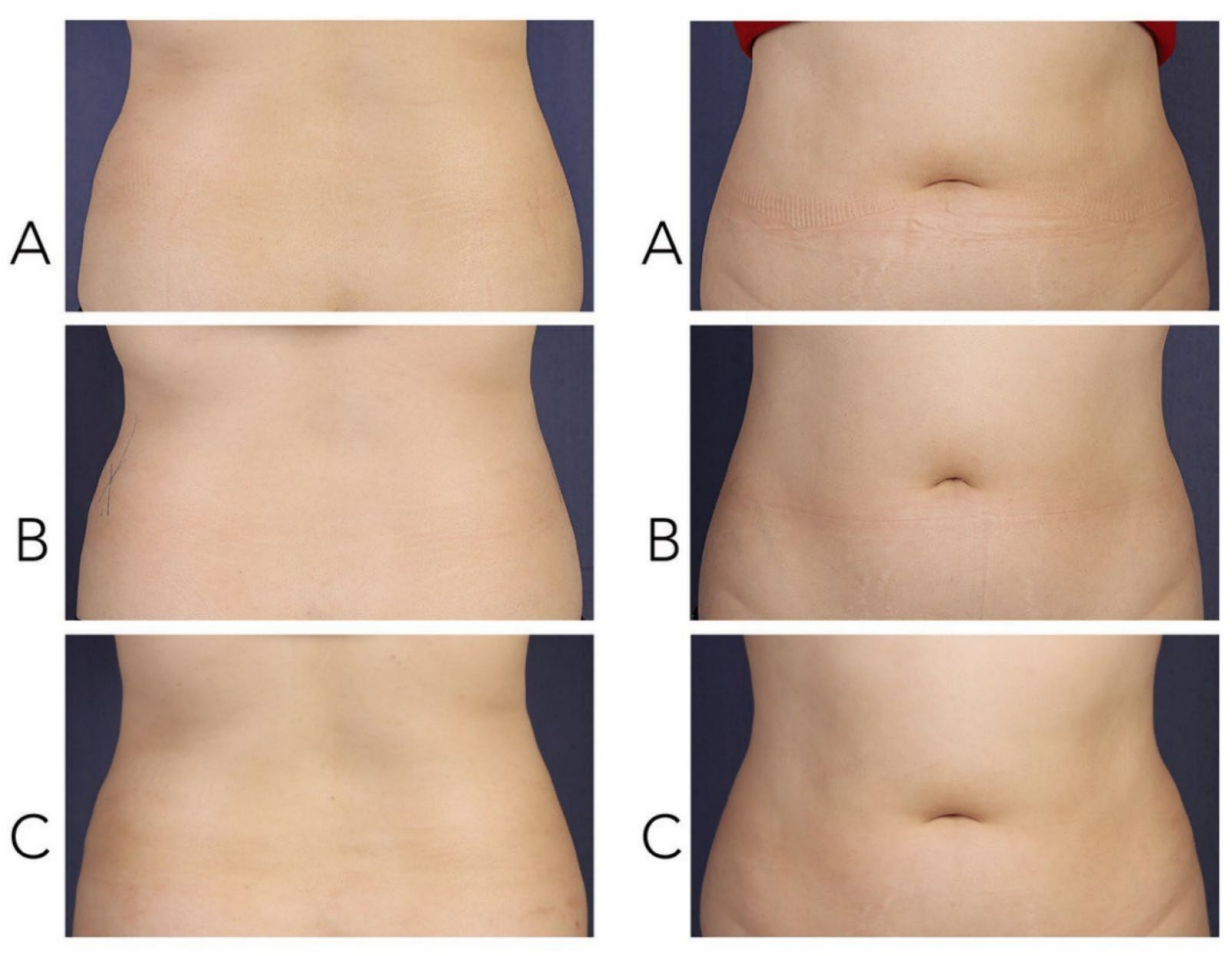
Standardized photography of anterior and posterior views: (A) baseline; (B) week 6; (C) week 12.

Two board‐certified dermatologists independently assessed the standardized photographs obtained at weeks 6 and 12 to evaluate aesthetic improvement. These were compared to baseline images using the Global Aesthetic Improvement Scale (GAIS), which scores changes as follows: +3 (very much improved), +2 (much improved), +1 (improved), 0 (no change), −1 (worse), −2 (much worse), and −3 (very much worse). Comparisons were made between images taken at Baseline, Week 6, and Week 12 (Figure 2).

### 2.4 Statistical Analysis

Quantitative variables were described using mean and standard deviation (SD) for normally distributed data, or median and interquartile range (IQR) for non‐normally distributed data. Categorical variables were summarized as frequencies and percentages. Changes over time were analyzed using repeated measures ANOVA or the Friedman test, depending on data distribution. Comparisons between baseline and other time points were conducted using the paired *t*‐test or the Wilcoxon signed‐rank test, as appropriate. A *p*‐value of < 0.05 was considered statistically significant. All analyses were performed using R software (version 4.4.3).

## 3 Results

A total of 30 participants were included in the study, with a predominance of female participants (86.7%). The mean age of the participants was 34.93 ± 4.83 years (Table 1).

**TABLE 1:** Descriptive statistics of the demographic variables.

Characteristic	*n* = 30
Gender, *n* (%)	
Female	26 (86.7%)
Male	4 (13.3%)
Age	
Range	25–45
Mean ± SD	34.93 ± 4.83

### 3.1 Efficacy Outcomes

Based on the Global Aesthetic Improvement Scale (GAIS), after the first injection, 70% (*n* = 21) of participants showed improvement compared to baseline, with 4 participants (13.3%) experiencing much improved and 17 participants (56.7%) as improved. Following the second injection, 86.7% (*n* = 26) of participants exhibited improvement compared to baseline, including 9 participants (30%) with much improved and 2 participants (6.7%) with very much improved. Notably, a comparison between aesthetic improvement at Week 12 and Week 6 by investigator‐assessed GAIS resulted in 17 participants (56.7%) with no change score, while 13 (43.3%) of participants still showed overall improved outcomes (Table 2).

**TABLE 2:** Descriptive frequency of GAIS index.

Characteristic	GAIS (baseline‐week 6) *n* = 30	GAIS (baseline‐week 12) *n* = 30	GAIS (week 6–week 12) *n* = 30
GAIS, *n* (%)			
No change	9 (30.0%)	4 (13.3%)	17 (56.7%)
Improved	17 (56.7%)	15 (50.0%)	12 (40.0%)
Much improved	4 (13.3%)	9 (30.0%)	1 (3.3%)
Very much improved	0 (0.0%)	2 (6.7%)	0 (0.0%)

The median waist circumference was 92.3 cm at baseline and 89 cm after the second session, which showed a statistically significant reduction over time (*p* < 0.001). The median body weight remained relatively stable throughout the study. No significant difference was found over time (*p* = 0.264).

No statistically significant changes were observed in thigh circumference over the follow‐up period. For the right thigh, the median circumference remained relatively stable across time points, with a slight decrease from baseline to Week 6, followed by a minor increase at Week 12 (*p* = 0.278). Similarly, the left thigh circumference demonstrated a modest fluctuation during the study period, with no significant overall trend detected (*p* = 0.061).

The mean right caliper thickness decreased significantly over time, measuring 4.89 ± 0.58 mm at baseline, 4.52 ± 0.62 mm after the first session, and 4.16 ± 0.59 mm after the second session, indicating a statistically significant decrease (*p* < 0.001). The left caliper thickness also significantly decreased, with a mean of 4.76 ± 0.59 mm at baseline and 4.03 ± 0.56 mm at week 12 (*p* < 0.001). (Table 3 presents a comparative analysis of waist circumference, body weight, thigh circumferences, and caliper measurements across the follow‐up time points).

**TABLE 3:** Comparison of waist, weight, thigh circumferences and caliper variables at follow‐up times.

	Baseline *n* = 30	Week 6 *n* = 30	Week 12 *n* = 30	*p*
Waist				
Mean ± SD	91.83 ± 6.66	90.72 ± 6.31	88.98 ± 6.2	< 0.001
Median (Q1, Q3)	92.3 (89.0, 95.0)	91.0 (88.0, 95.0]	89.0 (85.0, 93.0)	
Weight				
Mean ± SD	68.57 ± 9.32	68.90 ± 9.49	69.13 ± 9.24	0.264
Median (Q1, Q3)	66.5 (62.0, 75.0)	68.0 (62.0, 75.0)	66.5 (62.0, 74.0)	
Right thigh				
Mean ± SD	59.98 ± 3.65	59.82 ± 3.87	60.23 ± 3.37	0.278
Median (Q1, Q3)	60.0 (58.0, 62.0)	59.5 (58.0, 63.0)	59.5 (58.0, 62.5)	
Left thigh				
Mean ± SD	59.50 ± 3.74	59.03 ± 3.82	59.35 ± 3.75	0.061
Median (Q1, Q3)	58.3 (57.0, 62.0)	58.5 (56.0, 61.0)	58.8 (57.0, 62.0)	
Right caliper				
Mean ± SD	4.89 ± 0.58	4.52 ± 0.62	4.16 ± 0.59	< 0.001
Median (Q1, Q3)	4.9 (4.6, 5.4)	4.7 (4.0, 5.0)	4.2 (3.8, 4.6)	
Left caliper				
Mean ± SD	4.76 ± 0.59	4.43 ± 0.60	4.03 ± 0.56	< 0.001
Median (Q1, Q3)	4.9 (4.5, 5.2)	4.6 (4.1, 4.8)	4.1 (3.8, 4.4)	

Regarding the sonographic characteristics, both the right and left dermis demonstrated a statistically significant increase over time (*p* < 0.001), with a median baseline of 2.2 mm for both the right and left dermis and 2.5 mm and 2.8 mm at week 12 for the right and left dermis, respectively. The thickness of the hypodermis showed a significant decrease, with a mean baseline of 23.70 ± 6.35 mm for the right hypodermis and 24.68 ± 6.43 mm for the left hypodermis, and a mean of 20.00 ± 3.99 mm for the right hypodermis and 20.69 ± 4.46 mm for the left hypodermis at week 12, respectively (*p* < 0.001) (Table 4).

**TABLE 4:** Comparison of dermis and hypodermis variables at follow‐up times.

Characteristic	Baseline *n* = 30	Week 6 *n* = 30	Week 12 *n* = 30	*p* ^a^, ^b^
Right dermis				
Mean ± SD	2.12 ± 0.41	2.27 ± 0.36	2.44 ± 0.42	< 0.001^a^
Median (Q1, Q3)	2.2 (1.8, 2.3)	2.2 (2.0, 2.5)	2.5 (2.0, 2.6)	
Right hypodermis				
Mean ± SD	23.70 ± 6.35	20.87 ± 4.61	20.00 ± 3.99	< 0.001^b^
Median (Q1, Q3)	22.7 (19.3, 27.2)	20.3 (16.9, 24.6)	19.9 (16.8, 23.7)	
Left dermis				
Mean ± SD	2.25 ± 0.44	2.44 ± 0.48	2.73 ± 0.49	< 0.001^a^
Median (Q1, Q3)	2.2 (1.9, 2.7)	2.4 (2.1, 2.8)	2.8 (2.3, 3.0)	
Left hypodermis				
Mean ± SD	24.68 ± 6.43	22.25 ± 4.96	20.69 ± 4.46	< 0.001^b^
Median (Q1, Q3)	24.3 (21.4, 28.9)	22.2 (18.0, 25.2)	20.8 (17.6, 24.3)	

^a^ Friedman test.

^b^ Repeated Measure test.

Participant satisfaction was observed at follow‐up visits, indicating a positive response to the intervention. At Week 6, the median satisfaction score was 7.0 (interquartile range [IQR]: 6.0–8.0). At Week 12, the median satisfaction slightly increased to 7.5 (IQR: 5.0–9.0). These absolute values reflect a favorable level of satisfaction at both time points. Although satisfaction scores showed a minor numerical increase by Week 12, statistical comparison using the Wilcoxon test revealed no significant difference between the two visits (*p* = 0.894), suggesting that participant‐perceived satisfaction remained stable over time (Table 5).

**TABLE 5:** Comparison of satisfaction.

Characteristic	Week 6 *n* = 30	Week 12 *n* = 30	*p* ^a^
Satisfaction			
Median (Q1, Q3)	7.0 (6.0, 8.0)	7.5 (5.0, 9.0)	0.894
Mean ± SD	6.60 ± 2.74	6.50 ± 3.08	

^a^ Wilcoxon test.

### 3.2 Safety and Adverse Events

A total of 68 AEs were reported during the study period. The reported AEs occurred at the injection site, and most were mild to moderate in severity. These included injection site bruising (38.2%), pain (30.8%), swelling (14.7%), and pruritus (11.8%). In most cases, no specific medical intervention was required, and symptoms resolved spontaneously. When management was necessary, it was limited to applying cold compresses for swelling and analgesics for pain. Most AEs subsided within 2–3 days (Table 6).

**TABLE 6:** Descriptive frequency of adverse events.

Adverse events	*N* (%)
Injection site bruising	26 (38.2)
Injection site pain	21 (30.8)
Injection site swelling	10 (14.7)
Injection site pruritus	8 (11.8)
Injection site necrosis	1 (1.5)
Injection site urticaria	1 (1.5)
Injection site skin tightness	1 (1.5)

Rare injection site adverse events included urticaria, necrosis, and skin tightness, each reported in 1.5% of cases. One participant presented 1 week after the second injection session, complaining of exacerbated bruising and the presence of an ulcer. Upon examination, a superficial ulcer was identified. Given the potential for necrosis, the participant's medical history and treatment details were reviewed, revealing that among four 1 cc syringes used for the injection (two syringes for each side), one contained a needle shorter than the standard length and had been inadvertently used (0.5 cm instead of the standard 1.3 cm). Notably, a small area on one side of the injection site exhibited signs of bruising and ulceration. Consequently, this participant was diagnosed with superficial necrosis secondary to superficial injection. Supportive treatment was started, which included a topical antibiotic ointment and a wound healing agent, as well as 500 mg capsules of cephalexin administered every 6 h for 1 week. Additionally, the participant underwent two pulsed dye laser (PDL) therapy sessions at 2‐week intervals. By the end of the 8th week, only a patchy area of post inflammatory hyperpigmentation (PIH) remained at the site. This case highlights the need for standardized injection protocols to minimize the risk of such adverse events.

## 4 Discussion

In this single‐arm trial, we assessed the efficacy and safety of Embella (DCA injections, manufactured by Espad Pharmed Darou Company) for localized fat reduction in the flank region, utilizing both subjective and objective outcome measures. Following two treatment sessions, significant reductions were observed in waist circumference, caliper thickness, and hypodermis thickness, indicating a measurable effect on subcutaneous fat. Sonographic assessments revealed a statistically significant increase in dermal thickness, potentially reflecting localized inflammatory or reparative changes postinjection. However, thigh circumference and overall body weight remained unchanged, suggesting that DCA's lipolytic effects were localized and not systemic; this also indicates that the observed changes were not attributable to weight loss in the participants. GAIS ratings supported these findings, with 86.7% of participants showing improvement by the end of the study.

The treatment was generally well tolerated, with most AEs being mild to moderate and resolving without intervention. Common AEs included injection site bruising, pain, and swelling, aligning with known safety profiles of DCA. A single case of superficial necrosis was attributed to improper injection technique and resolved with conservative management. Participant satisfaction remained consistently favorable throughout the follow‐up period, reinforcing the clinical relevance of the observed objective outcomes. Overall, the results support the localized efficacy and acceptable safety profile of DCA injections for body contouring in the flank area.

DCA is an FDA‐approved injectable therapy that has been the first approved medical treatment for reducing submental fat since 2015. This treatment has gained popularity due to its high efficacy, ease of use, bearable side effects, and limited individual downtime [6, 7]. However, in recent years, it has also been used off‐label for body contouring in various areas, including the jowls, back, abdomen, and arms [6, 8].

DCA is a secondary bile acid produced by the gut microbiome that plays a crucial role in the emulsification and solubilization of fats. Lipolysis occurs through the disruption of the adipocyte membrane, leading to cell lysis. This process triggers a local inflammatory response aimed at eliminating cellular and fat debris via macrophages. Additionally, fibroblast‐mediated neocollagenesis occurs. However, no significant changes were observed in the systemic concentrations of lipid profiles, such as total cholesterol and triglycerides, or in adipokines, including IL‐6, IL‐15, and TNF‐α [8, 9, 10, 11].

When injecting DCA, subcutaneous fat and protein‐poor tissues are primarily affected by cytolysis, while protein‐rich tissues such as skin, muscles, and blood vessels remain largely unaffected due to the attenuation of DCA's cytolytic activity by albumin [10, 11]. A recently published study indicates that, in addition to the lipolytic effects of DCA, it significantly reduces fat mass and enhances both fat and carbohydrate metabolism. Furthermore, improvements in glucose metabolism were observed, leading to enhanced glucose tolerance and insulin sensitivity in this mouse model study [12].

Our study demonstrated that conducting two sessions of injections for flank fat reduction can significantly impact the fat volume in adjacent areas; however, it does not affect remote areas, such as thigh circumference, as observed in our study. Furthermore, no significant weight changes were noted among the participants, indicating that the fat reduction is specifically related to the injection of DCA.

Like this study, Salvador et al. conducted research in which DCA was injected into four distinct areas of the abdomen: the upper right, lower right, upper left, and lower left hypogastric regions. The final results were assessed using caliper measurements, ultrasonography, and three‐dimensional scan techniques. In contrast to our study, they reported varying results in fat reduction across the measured quadrants, particularly concerning caliper and sonographic measurements [13].

A separate study evaluated the efficacy of DCA injection in reducing abdominal fat and proposed its mechanism through the increased formation of crown‐like structures, macrophage infiltration, and decreased expression of leptin, hormone‐sensitive lipase, triglyceride lipase, and CD36, ultimately resulting in fat tissue necrosis. No significant changes were observed in systemic inflammatory markers such as C‐reactive protein (CRP), lipid profile, or blood glucose levels, suggesting that DCA primarily exerts localized effects without a systemic metabolic impact [14].

The correct use of DCA is essential for achieving effective and safe DCA injections. In this context, the time intervals between sessions and the volume of injections are critical factors. Both of these elements are influenced by subject preferences, treatment costs, downtime, and anatomical and physiological considerations, such as fat distribution in the treated area [15, 16]. Since there have not been many studies conducted on DCA injections in abdominal regions, most reported AEs pertain to complications arising from submental fat injections. However, skin reaction complications are commonly observed across various body areas [7, 15].

Bruising and pain at the injection site were among the most frequently reported AEs in previous studies, which aligns with the findings of this study (38.2% and 30.8%, respectively) [15, 16]. Edema and induration are other common complications easily managed with ice packs, anesthetics, and analgesics [7, 17].

A rare adverse event observed in one individual in this study was necrosis at the injection site, which has only been reported in the context of submental fat injections of DCA to date. It is believed that the occurrence of necrosis may result from intradermal injection. Aside from this case, no severe AEs were reported during the trial.

This study presents valuable insights into using DCA injections for flank fat reduction; however, several limitations must be acknowledged. First, the relatively small sample size may limit the generalizability of the findings. Second, the absence of a control group prevents comparison with natural variation or placebo effects, thereby limiting causal inferences. Third, while combining standard photography, caliper measurements, ultrasonography, and anthropometric data (thigh circumferences and BMI) enhances objectivity, potential measurement bias and interobserver variability cannot be excluded, especially in manual techniques such as caliper assessment. Additionally, although BMI was monitored to ensure weight stability, body composition changes (e.g., muscle mass) were not assessed, which may influence the interpretation of fat volume reduction. Finally, the 12‐week follow‐up period may not adequately capture long‐term treatment durability or delayed adverse events. Future studies should include larger, randomized cohorts with longer follow‐ups, more precise imaging tools (e.g., Magnetic resonance imaging (MRI)), and subject‐reported outcomes better to evaluate efficacy, durability, and participant satisfaction.

## 5 Conclusion

DCA injections, Embella (manufactured by Espad Pharmed Darou Company), showed effective localized fat reduction in the flank area after two sessions, with high rates of overall aesthetic improvement appraised by GAIS, and favorable patient satisfaction scores. It also demonstrated significant changes in ultrasound and caliper measurements. AEs were mostly mild to moderate and manageable, though one case of superficial necrosis highlights the importance of proper technique. No serious reactions were reported in this study.

## Author Contributions

N.N.E. was responsible for the conceptualization, methodology design, manuscript writing, and data visualization. F.K., A.E., M.N. and H.M. contributed to data curation, investigation, provided supervision, and participated in the review and refinement of the methodology. H.K., A.S. and K.S. contributed to study coordination and logistical planning and provided general scientific input during early stages of the project. H.G. was responsible for the research and development (R&D) and production of the investigational product. H.K., A.S. and H.G. did not participate in data collection, data interpretation, statistical analysis, or the writing of the results and discussion sections. K.B. contributed to the conceptual framework, supervised the project, supported methodological development, contributed to visualization, and reviewed and edited the manuscript. The scientific team conducted all data analysis and interpretation independently to preserve the neutrality of the study outcomes.

## Conflicts of Interest

Hamidreza Kafi is Head of Medical Department of Orchid Pharmed Company which is in collaboration with Espad Pharmed Darou Company with respect to conducting clinical trials. Araz Sabzvari is a member of CinnaGen Medical Biotechnology Research Center, which collaborates with universities and researchers all over the world with regards to research and development of medications and health issues. Hoshyar Gholami is the Chief Executive Officer of Espad Pharmed Darou Company and has been directly involved in the research and development (R&D) and production of the product used in this study. While the company provided funding for the study, all data collection, analysis, and interpretation were conducted independently by the research team to ensure objectivity and minimize potential bias. Other authors declare no conflicts of interest.

## Data Availability

The data that support the findings of this study are available from the corresponding author upon reasonable request.
